# Supplementary material for: Fast Responses of Root Dynamics to Increased Snow Deposition and Summer Air Temperature in an Arctic Wetland
Source: Front Plant Sci. 2018 Aug 30;9:1258. doi: 10.3389/fpls.2018.01258 (PMC6125414; doi:10.3389/fpls.2018.01258)
Supplement: Supplementary file 1 [file Data_Sheet_1.docx]

Supplementary Material

Fast responses of root dynamics to increased snow deposition and summer air temperature in an arctic wetland

**Ludovica D’Imperio^*^, Marie Frost Arndal, Cecilie Skov Nielsen, Bo Elberling, Inger Kappel Schmidt**

*** Correspondence:**

Ludovica D’Imperio, Center for Permafrost (CENPERM)

[ldi@ign.ku.dk](mailto:ldi@ign.ku.dk)

**Supplementary Figure**
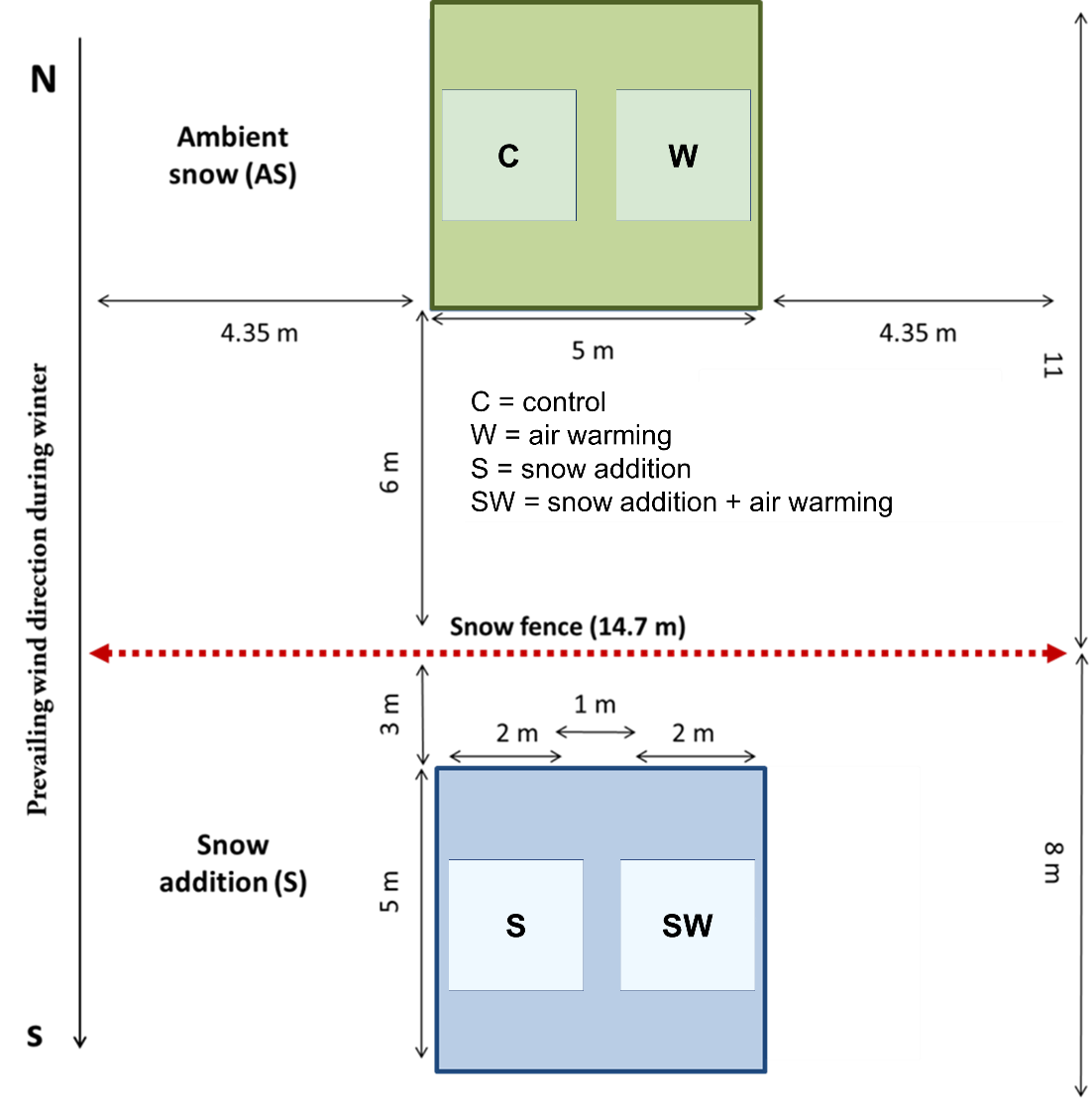
**1.** Example of snow fence installed at the site in 2013. The sketch represents a unit block split by a snow fence built against the prevailing wind direction during wintertime. The green plots at the windward side of the snow fence are exposed to “ambient snow” and include control (C) and air warming (W) by open top chambers (OTCs). The blue plots at the leeward side of the snow fence are exposed to passive “snow addition” and include snow addition (S) and the combination of snow addition + air warming with OTCs (SW).


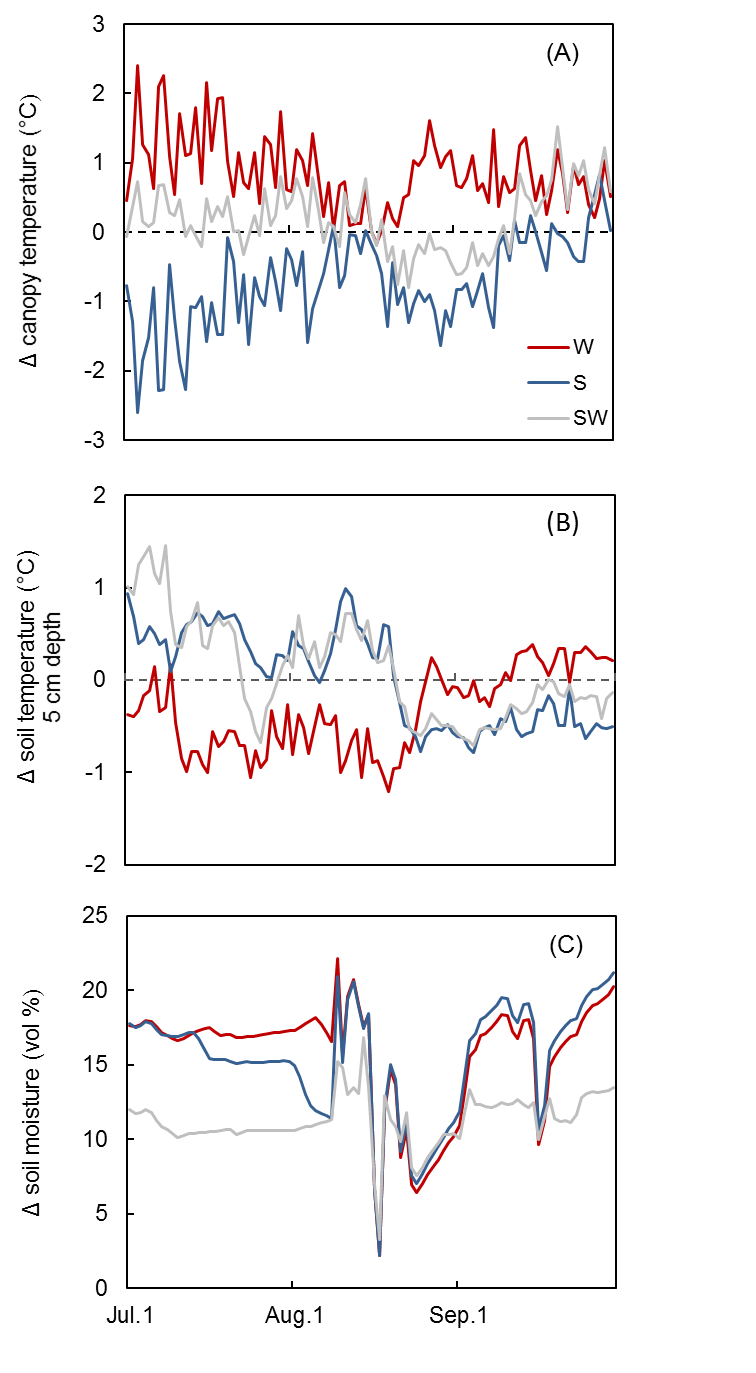


**Supplementary Figure 2.** Differences between single treatments and controls in daily averages of continuous measurements of A) Air temperature within the canopy (2 cm above ground) B) soil temperature and C) soil moisture recorded at the site during the growing season in 2014 (n=3). In legend: warming by OTCs (W), snow accumulation (S) and snow + warming by OTCs (SW).

**Supplementary Table 1.** Timeline of the installations and measurements done at the site in Blæsedalen during the course of this study.

| Year | Treatment | Years since installation | Measurement / experiment | Methodology |
| --- | --- | --- | --- | --- |
| 2013 | Warming  Snow  Snow + warming | - | Snow fences installation,  ingrowth bags ^a^ and minirhizotrons | Soil cores |
| 2014 | Warming  Snow  Snow + warming | 1 | Root images/growth  Root production  Root turnover | Minirhizotrons  Ingrowth bags |

^a^ The ingrowth bags were installed only at the plots with “snow” as main treatment.

**Supplementary Table 2.** Average single root length during each measurement campaign in all treatment plots (mean ±SE). Treatment abbreviations stand for: control (C), warming with OTCs (W), snow accumulation (S) and snow + warming with OTCs (SW).

| Date (2014) | Treatment | Root length (cm) | ±SE | Root  length ^a^ (mm cm^-2^) | ±SE |
| --- | --- | --- | --- | --- | --- |
| 2 Jul | C | 3.00 | 0.48 | 0.044 | 0.008 |
|  | W | 2.31 | 0.24 | 0.025 | 0.003 |
|  | S | 2.38 | 0.39 | 0.037 | 0.006 |
|  | SW | 3.36 | 0.77 | 0.035 | 0.008 |
| 24 Jul | C | 2.71 | 0.41 | 0.040 | 0.007 |
|  | W | 2.27 | 0.31 | 0.029 | 0.007 |
|  | S | 1.71 | 0.18 | 0.019 | 0.003 |
|  | SW | 2.12 | 0.07 | 0.022 | 0.001 |
| 13 Aug | C | 2.72 | 0.36 | 0.038 | 0.003 |
|  | W | 2.29 | 0.36 | 0.029 | 0.007 |
|  | S | 1.82 | 0.21 | 0.021 | 0.004 |
|  | SW | 2.06 | 0.17 | 0.022 | 0.002 |
| 8 Sep | C | 2.72 | 0.32 | 0.036 | 0.002 |
|  | W | 2.34 | 0.31 | 0.030 | 0.006 |
|  | S | 1.89 | 0.21 | 0.022 | 0.004 |
|  | SW | 2.16 | 0.16 | 0.023 | 0.002 |
| 17 Sep | C | 2.78 | 0.35 | 0.036 | 0.003 |
|  | W | 2.28 | 0.37 | 0.031 | 0.008 |
|  | S | 1.95 | 0.24 | 0.023 | 0.005 |
|  | SW | 2.22 | 0.19 | 0.023 | 0.002 |

^a^ Averaged root length estimated per tube area

**Supplementary Table 3.** Average single root length at different soil depths in all treatment plots (mean ± SE) during the growing season in 2014. Treatment abbreviations stand for: control (C), warming with OTCs (W), snow accumulation (S) and snow + warming with OTCs (SW).

| Soil depth (cm) | Treatment | Root  length  (cm) | ±SE | Root  length  (mm cm^-2^) | ±SE |
| --- | --- | --- | --- | --- | --- |
| 0-10 | C | 2.26 | 0.09 | 0.11 | 0.004 |
|  | W | 1.78 | 0.06 | 0.09 | 0.003 |
|  | S | 2.19 | 0.07 | 0.11 | 0.004 |
|  | SW | 1.99 | 0.07 | 0.10 | 0.003 |
| 10-20 | C | 2.34 | 0.08 | 0.11 | 0.004 |
|  | W | 2.26 | 0.08 | 0.11 | 0.004 |
|  | S | 1.19 | 0.05 | 0.06 | 0.002 |
|  | SW | 1.98 | 0.06 | 0.10 | 0.003 |
| 20-30 | C | 3.55 | 0.16 | 0.17 | 0.008 |
|  | W | 2.35 | 0.09 | 0.11 | 0.004 |
|  | S | 2.17 | 0.13 | 0.10 | 0.006 |
|  | SW | 2.63 | 0.11 | 0.13 | 0.005 |
| 30-40 | C | 3.46 | 0.26 | 0.17 | 0.013 |
|  | W | 3.38 | 0.16 | 0.16 | 0.008 |
|  | S | 2.64 | 0.19 | 0.13 | 0.009 |
|  | SW | 2.24 | 0.11 | 0.11 | 0.005 |
